# Assessing Entrepreneurial Characteristics of Healthcare Students Participating in an Entrepreneurial-Medicine Curriculum

**DOI:** 10.15694/mep.2018.0000101.1

**Published:** 2018-05-15

**Authors:** Alexander Watson, Camden MacDowell, Gregg Khodorov, Julia Tartaglia, Paul Weber

**Affiliations:** 1 Rutgers-Robert Wood Johnson Medical School

**Keywords:** Medical education, Entrepreneurship education, Healthcare innovation, Innovation curriculum

## Abstract

This article was migrated. The article was not marked as recommended.

As innovation transforms the healthcare industry, medical institutions are increasingly incorporating business skills and concepts into their curricula. The goal of this study was to characterize the types of students who engage in these supplemental curricula with respect to four entrepreneurial characteristics: entrepreneurial interest, support, confidence, and intention. We sampled students participating in a healthcare Innovation Summit using a validated survey to assess these characteristics. The sampled population reported significantly positive Interest and Support regarding an entrepreneurial career (5.18 and 5.80; p-values <0.01), whereas Intention and Confidence did not significantly differ from neutral (4.02 and 3.78; p>0.05). Individuals’ entrepreneurial Interest was significantly positively correlated with all other characteristics; demonstrated by Pearson’s Correlation Coefficients of 0.74, 0.62, and 0.59 when comparing sample means of Interest versus Intention, Confidence, and Support, (p-values <0.05). In addition, significant correlation between Intention and Confidence was observed (Pearson’s R = 0.78: p-values <0.05). Conversely, sample means for entrepreneurial Support were not significantly correlated with either Intent or Confidence (p-values >0.05).

Our findings supply foundational data for understanding the growing cohort of individuals engaging in entrepreneurial-medicine. These data demonstrate the integrated nature of various entrepreneurial characteristics in these populations and support the idea that investment, which promotes one area, would translate to increases in others.

## Introduction

The growth of digital health innovations, rapid advances in personalized medicine, and new frontiers for biotechnology are disrupting the way healthcare is being practiced and impacting all areas of the industry. In 2017 alone, investments in digital health surpassed $11.5 Billion, up from $8.2 Billion in 2016 ( Hanin P.; Dordai 2018). In order for physicians to engage with these rapid innovations in the healthcare ecosystem, both medical schools and healthcare industry professionals realize the need for physicians to supplement their clinical knowledge with skills and techniques from the business sector ( Gonzalo et al. 2017). Current medical trainees also seem to realize this need and desire to improve their entrepreneurial skill sets, as demonstrated by a recent survey of 1,299 medical students at 18 US medical schools where nearly two thirds (65.2%) of students reported being interested in learning more about the business aspects of medicine ( Wanke et al. 2015).

One way to receive this medical-entrepreneurship training is through MD-MBA combined degree programs, and the number of such programs in the US has increased dramatically in recent decades, from less than 10 in 1993 to over 60 today ( Goyal et al. 2015). These combined-degree programs come with caveats - namely, additional years and tuition on top of students’ already long and expensive medical training. In an effort to better equip future physicians to solve healthcare’s complex problems without pursuing an additional professional degree, medical institutions are redesigning their curricula to allow students to learn about innovation and entrepreneurship alongside their medical education. In fact, at least 13 US allopathic medical schools have developed innovation and entrepreneurship programs to teach medical students about business, entrepreneurship, leadership, and technology as a supplement to their core clinical curriculum ( Niccum et al. 2017). Furthermore, several medical schools such as Rutgers-Robert Wood Johnson Medical School, University of Michigan Medical School and University of Pennsylvania’s Perelman School of Medicine have student-led organizations that create programming and experiential opportunities for medical students to learn about medical entrepreneurship.

A common type of teaching event used in these entrepreneurial curricula is a day-long “Innovation Summit.” These events bring together leaders from different fields of healthcare innovation to lecture on their area of expertise and describe the steps they took to bring their innovation to market. Throughout the day, participants engage with these speakers and are taught entrepreneurial techniques through panel and round table discussions, question & answer sessions, and skills seminars. Additionally, these summits may include opportunities for participants to interact with other attendees in workshops where they directly apply the basic business techniques they have learned in the preceding lectures. In this way, Innovation Summits and similar events are designed to simultaneously teach students entrepreneurial skills, impassion them to become healthcare innovators, and foster support networks of like-minded students and collaborators.

While these Innovation Summits and similar entrepreneurial curricula are becoming increasingly infused into healthcare training, there is a substantial gap in foundational knowledge about the types of students who attend these events and their sentiments regarding various aspects of entrepreneurship. Not only is this information critical to the success and improvement of these programs, but also such knowledge would enable tailoring of the agendas and material delivered at these events to best foster the entrepreneurial characteristics of the attending audience.

In addition to informing proper Innovation Summit event structure and planning, identifying specific characteristics of event participants provides evidence-based knowledge that institutions and student groups need in order to advocate for public and private funding to further sustain and enhance these programs. As a result, data demonstrating that students are actively interested in entrepreneurship and innovation can bolster the rationale for universities, local and state governments, and private investors to support entrepreneurial students and drive innovation forward. These data also have the potential to illuminate self-identified weaknesses in attendees’ expectations of success, such as knowledge, support, or confidence, which could then be targeted in future events.

Finally, this collected attendee data, particularly demographics data, can facilitate identification of any disparities between groups participating in these events. This, in turn, can highlight opportunities to promote entrepreneurial interest across student communities to help bolster diversity in the entrepreneurial-medicine sector.

In this research, we attempted to address this knowledge gap by quantifying the entrepreneurial characteristics of students attending a medical entrepreneurship event. To our knowledge, this is the first study that addresses these questions. As such, this study provides important foundational insights and serves as a baseline metric for future ‘trend’ studies analyzing the entrepreneurial features of similar event attendee populations over time.

### Purpose

The following study aimed to assess specific entrepreneurial characteristics of undergraduate and graduate student participants of a healthcare innovation and entrepreneurship summit. Our goal was to better understand the types of individuals who are attracted to such programming, their current intent to pursue a career involving entrepreneurship, and their sentiment toward various entrepreneurial characteristics (e.g. entrepreneurial interest, support, intent, and confidence: terms defined in **Methodology**).

Our intention is to facilitate understanding of event attendee characteristics and their relationships to each other (e.g. do certain entrepreneurial characteristics trend together?). Ideally, these insights will enable institutions to 1) better tailor entrepreneurial curricula to support the characteristics of individuals that participate in these events 2) encourage further grant funding and support to foster these entrepreneurial characteristics in their students and 3) promote interest in medical entrepreneurship across diverse student groups. Furthermore, given this study’s objective as a foundation for future investigations into entrepreneurial trends in similar populations, we assessed whether the four entrepreneurial metrics measured here were sufficient to capture the entire entrepreneurial landscape of the sampled population. These data will help to inform whether future studies should include additional measurement metrics.

We conducted this study by surveying participants of the Rutgers Biomedical Entrepreneurship Network (BEN) Healthcare Innovation Summit, an event that is part of the supplemental entrepreneurial-medicine curriculum at Rutgers-Robert Wood Johnson Medical School. The event was open to the public and attracted 123 attendees, of which 59 were medical, graduate, and undergraduate students. We hypothesized that the students participating in this event would exhibit significantly positive levels of interest, confidence, support, and intent of engaging in entrepreneurial careers.

## Methodology

### Setting

The BEN Healthcare Innovation Summit was a one-day event open to the public featuring keynote presentations from industry professionals on topics including the latest innovations in healthcare delivery, insurance, medical education, telemedicine, biotechnology, health information technology, and healthcare venture capital. The event included an experiential learning workshop accompanied by small-group, roundtable discussions. The Innovation Summit took place on Saturday, April 1, 2017 in The Arline & Henry Schwartzman Courtyard at Robert Wood Johnson University Hospital in New Brunswick, New Jersey.

### Population

This study was approved by the Rutgers New Brunswick Health Sciences Institutional Review Board - study number Pro20170000012. Participants of this survey-based study consisted of registrants for the Innovation Summit. Potential respondents were screened for eligibility at the start of the survey. In order to be eligible to complete the study, participants had to be registered to attend the summit and currently enrolled as an undergraduate or graduate student within New Jersey. Participants were not compensated for their response. The final sample consisted of 21 undergraduate and graduate students.

### Recruitment and Data Collection

Participants were invited to partake in the survey during the online registration process. After registering for the event, individuals were redirected to a description of the survey and provided with a link to access the survey instrument. This link was also provided at check-in on the day of the event to qualifying attendees. These eligible conference attendees could opt to take the survey via an iPad at the registration table on the day of the conference or on their own personal device.

### Survey Questionnaire

We used a survey instrument developed and validated for assessing entrepreneurial features by do Paco et al( do Paço et al. 2011) to assess the following four characteristics:

• *Entrepreneurial Interes*t: a respondent’s attitude and orientation towards a future entrepreneurial career.
• *Support Network*: a respondent’s impression of the support/approval they would receive from people in their close environment if they pursued an entrepreneurial career.
• *Entrepreneurial Confidence*: a respondent’s confidence in their own entrepreneurial capacity, skills, and knowledge-base.
• *Entrepreneurial Intention*: a respondent’s intention to become an entrepreneur.

The complete survey questionnaire can be found in **Appendix 1**. For each survey question, respondents could select one of seven Likert Scale values describing their agreement with a pro-entrepreneurial statement ranging from 1 (complete disagreement/negative sentiment) to 4 (neutral) to 7 (complete agreement/positive sentiment). These scales meet adequate measures for composite reliability and discriminant validity ( do Paço et al. 2011), and thus provide an accurate assessment of the entrepreneurial characteristics of the population sample surveyed here.

### Data Analysis

All data analyses were performed using MATLAB (Mathworks, Natwick MA) and Excel (Microscoft, Redmond WA). Multiple survey questions addressed the same entrepreneurial characteristics, so respondents’ individual question scores were binned for each characteristic and the mean value calculated, resulting in a single summary score of 1-to-7 for each of the four entrepreneurial characteristics per respondent. Linear regression models comparing entrepreneurial characteristics were built using a least-squares fit. For multiple regression models all R ^2^ coefficients of best fit were adjusted to account for the multiple predictor variables.

## Results

In total, 21 surveys were received from the 77 eligible Innovation Summit participants. Two of these questionnaires were removed due to incomplete response, resulting in 19 completed surveys for analysis and a response rate of approximately 25%. A complete description of survey respondent demographics is presented in **Table 1**. Multiple ages, sex, marital status, education levels, and ethnicities were represented with a majority of respondents reporting as 21-to-25 year-old, single, Caucasian, male medical students.

Table 1.

**Figure:**
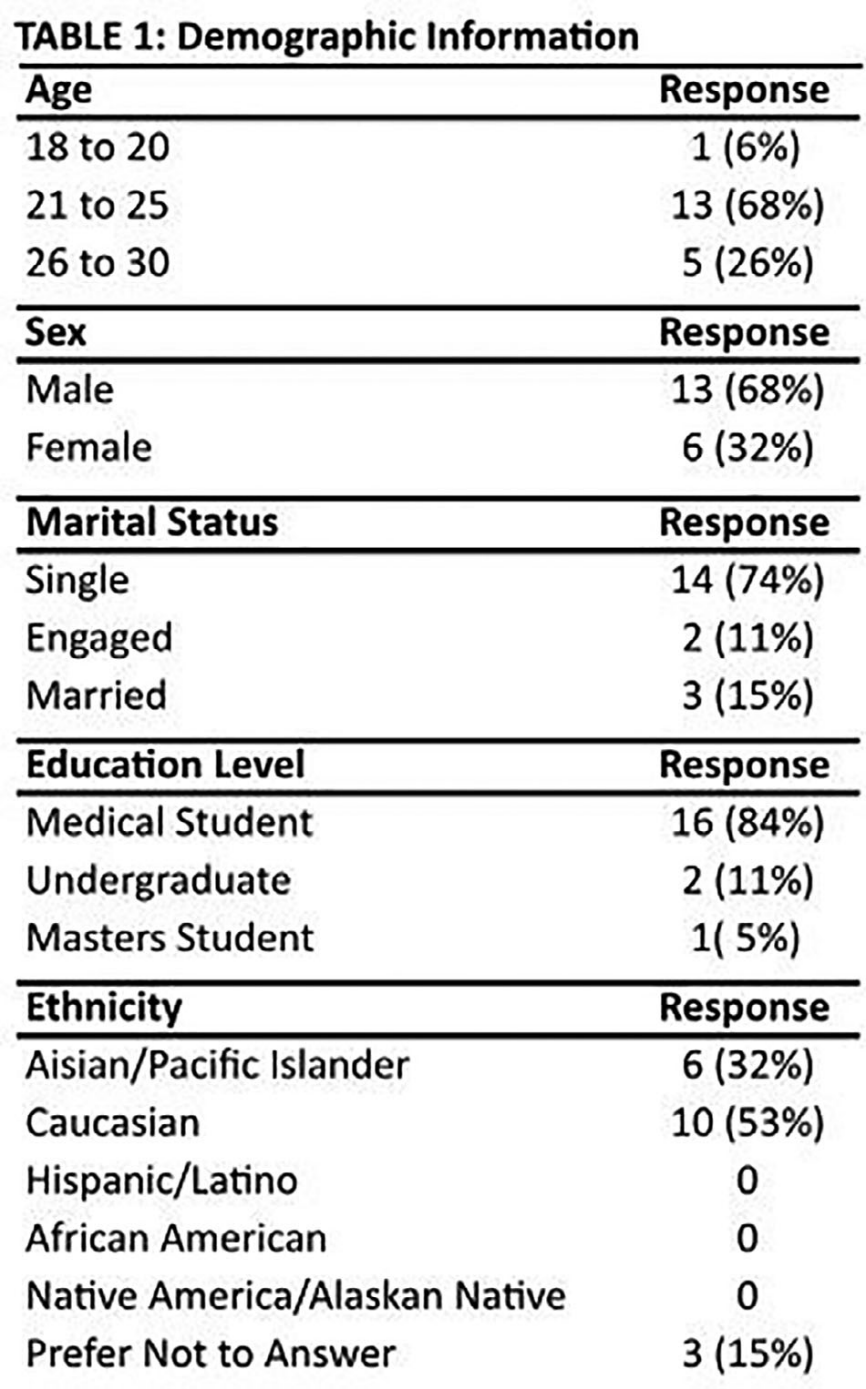


A complete list of the mean and standard deviation of responses to individual survey questions can be found in **Appendix 2**. **Figure 1** depicts the distribution of summary sentiment scores for each of the four combined entrepreneurial domains: *Interest, Support, Confidence,* and *Intent.* Overall, most characteristics had relatively wide score distributions, with all except *Support* containing both negative and positive sentiment values. *Support* had both the smallest distribution range (whisker-to-whisker: black dotted lines) and interquartile range (IQR: blue box) with values of 3 and 1.17 respectively. *Intention* had both the largest distribution and IQR at 6 and 2.25, respectively. A complete set of summary statistics can be found in the corresponding table of **Figure 1**.

**Figure:**
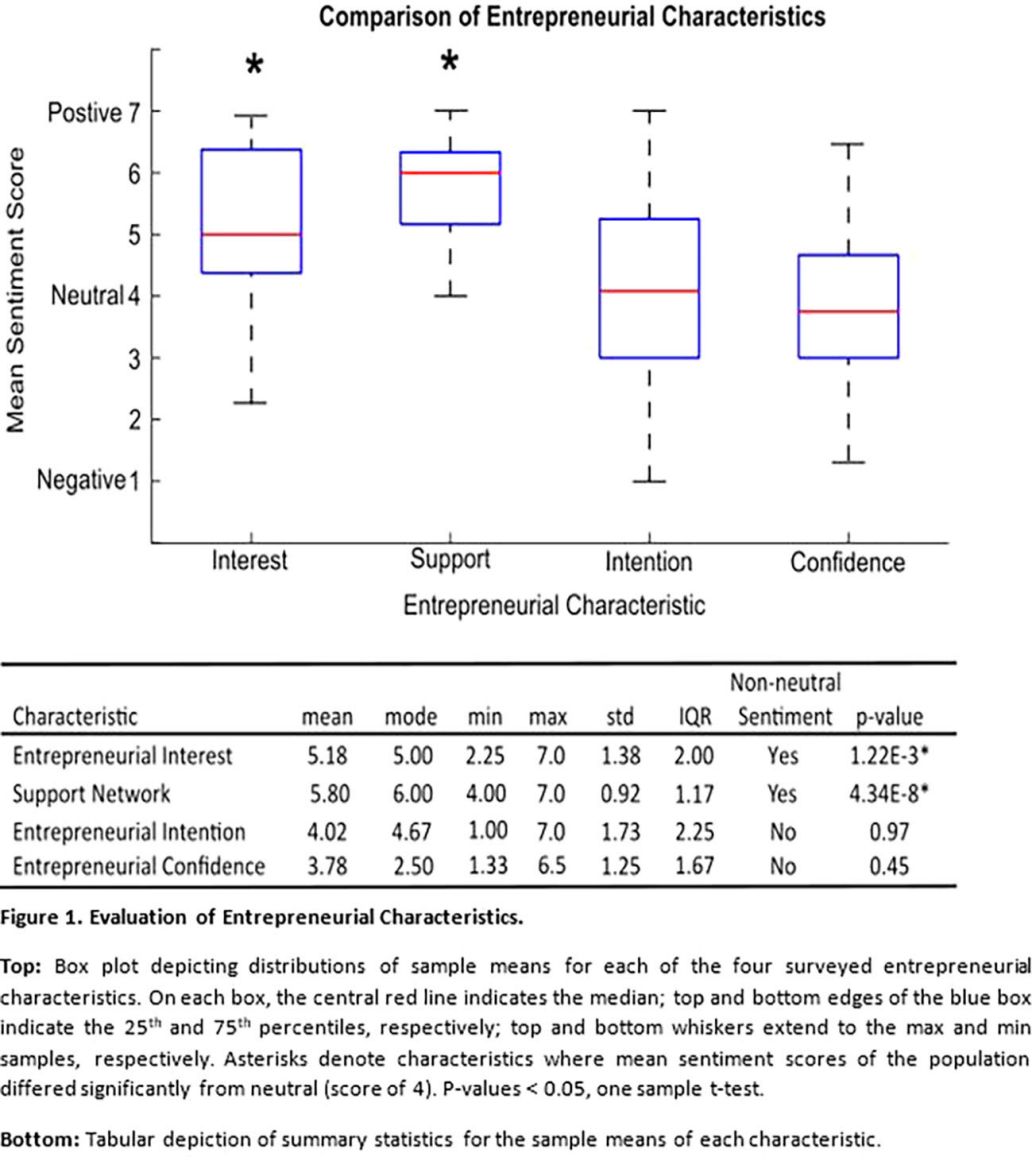


The sampled population of Innovation Summit participants reported a significantly positive *Interest* and *Support* sentiment towards an entrepreneurial career, calculated by one-sample t-tests comparing each characteristic’s mean score to a null hypothesis value of 4 (neutral sentiment). The mean *Intention* was near-neutral at 4.02. The mean *Confidence* sentiment score was slightly negative at 3.78, but did not significantly differ from neutral.

Next, we performed simple linear regression analyses to better understand the relationship between entrepreneurial characteristics. Specifically, we made comparisons between each of the six possible pairs of characteristics to identify possible trends between scores. **Figure 2** depicts the resulting analyses.

**Figure:**
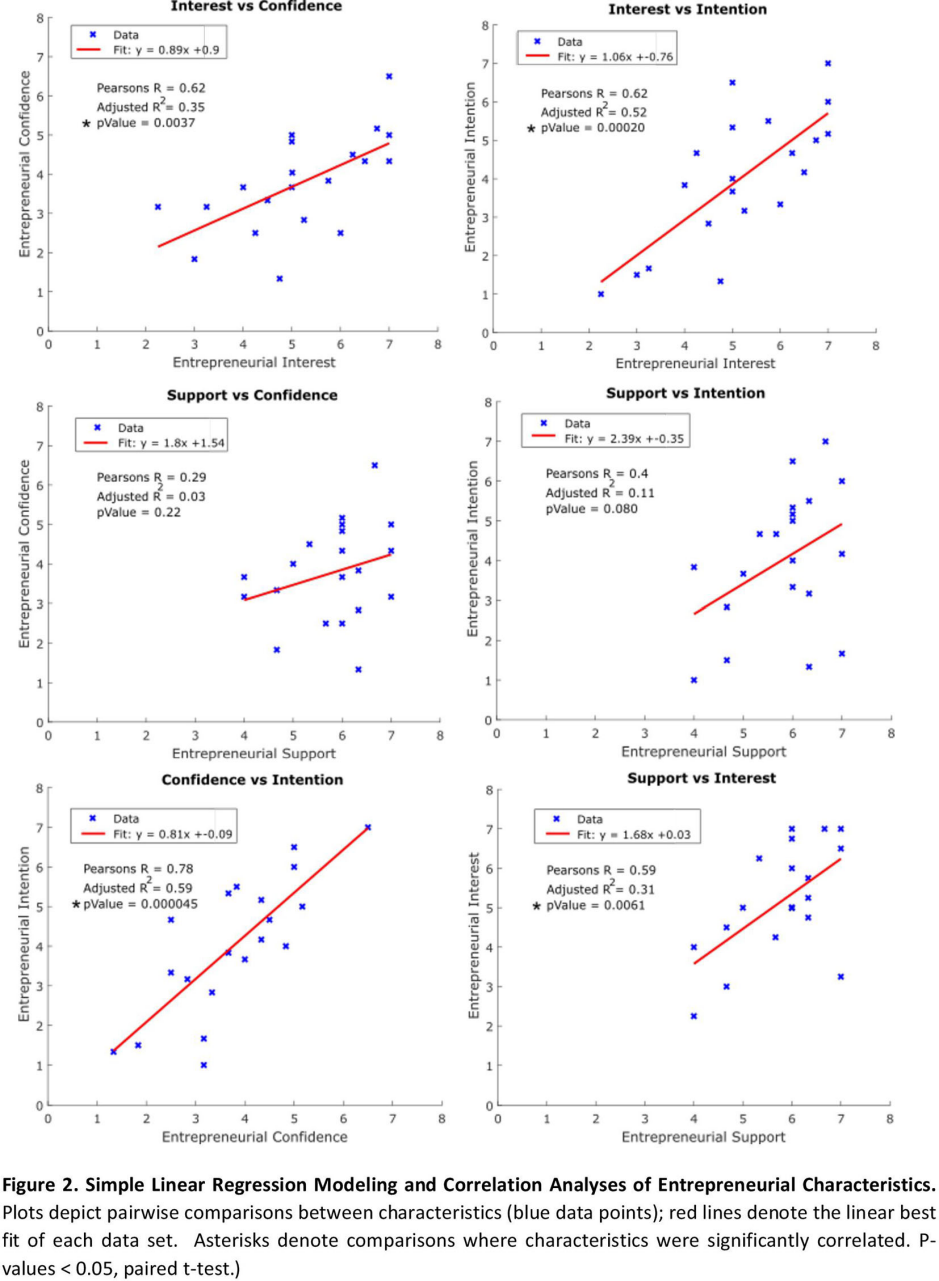


Individuals’ entrepreneurial *Interest* was significantly positively correlated with all three other characteristics; demonstrated by Pearson’s Correlation Coefficients of 0.74, 0.62, and 0.59 when comparing sample means of *Interest* versus *Intention*, *Confidence,* and *Support,* respectively (p-values <0.05).> *Intention* and *Confidence* was observed (Pearson’s R = 0.78: p-values <0.05).> *Support* were not significantly correlated with either *Intent* or *Confidence* (p-values > 0.05).

As discussed above, the primary focus of our analyses was to measure the relative distribution of sentiment regarding four entrepreneurial characteristics in Innovation Summit participants and assesses correlations between these characteristics, objectives accomplished above in **Figures 1** and **2**. However, as shown in **Figure 2** the adjusted R-squared values for these pairwise comparisons were relatively low, which indicates that simple bivariate linear models were not sufficient to accurately capture the observed variability in the data. As such, a secondary analysis was performed to determine if, collectively, the variables surveyed in this study accurately captured the variability in the widely distributed and neutral-centered *Confidence* and *Intention* distributions, or whether additional variables may need to be assessed in future studies. Multiple linear regression analyses were performed, modeling *Confidence* and *Intent* each as functions of the remaining three variables ( **Figure 3**). These multiple regression models more accurately captured the observed variability in *Intention* and *Confidence* with resulting adjusted R-squared values of 0.66 and 0.55, respectively.

**Figure:**
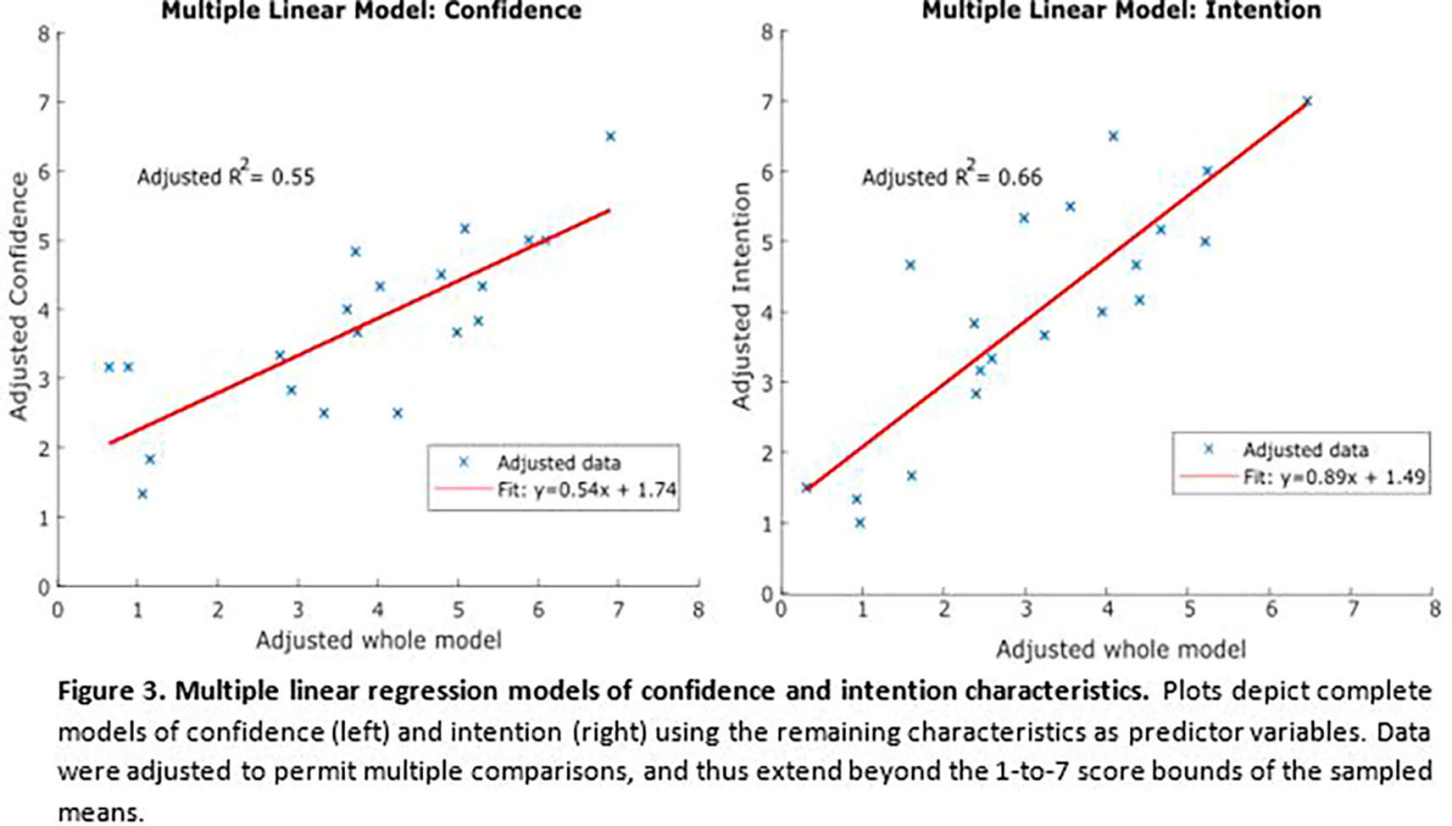


## Discussion

As innovation and entrepreneurship continue to transform healthcare, medical institutions are increasingly incorporating business skills and knowledge into their curricula. The goal of this study was to understand the types of students that engage in these curricula by assessing the entrepreneurial characteristics of students participating in a business and healthcare innovation summit.

As suggested by the total number of attendees at the event (123 people), passive interest in medical-entrepreneurship likely exists amongst students, faculty, and other members of the healthcare industry. Almost 50% of these attendees were undergraduate, graduate, or medical students; data that underscores the increasing interest in entrepreneurship in these populations which has been well-documented in previous studies ( Wanke et al. 2015).

Given that these students were participating in a voluntary, supplemental entrepreneurial curriculum event, we hypothesized that the sampled population would exhibit significantly positive mean responses for all four measured entrepreneurial characteristics. However, this was not the case. Rather, while respondents reported significantly positive attitudes towards a future entrepreneurial career (e.g. *Interest*) and perceived positive support from their community and peers for entrepreneurial pursuits (e.g. *Support*), they were neutral regarding both their *Confidence* in their entrepreneurial abilities and *Intention* to pursue an entrepreneurial venture ( **Figure 1**).

It is important to acknowledge the significant, positive relationship between entrepreneurial *Interest* with each of the other characteristics ( **Figure 2**). This suggests that - as one would expect - overall excitement towards entrepreneurship is critical to a student’s confidence, perceived support, and intention towards an entrepreneurial pursuit. Conversely, the lack of significant correlations between *Support* and *Intention* or *Support* and *Confidence* suggests that peer and faculty support alone is not sufficient to engage these students in entrepreneurial pursuits. Rather, our results suggest there’s likely a complex interplay between an individual’s various entrepreneurial characteristics. Taken together, these data imply that multi-faceted curricula targeting multiple domains of entrepreneurship (such as Innovation Summit-type events) may be the most effective means of fostering innovative pursuits in these populations.

The results of this study provide foundational data for institutions and student groups advocating for grant funding for supplemental medical-entrepreneurship curricula. The significantly positive *Interest* and *Support* in this sample ( **Figure 1**) suggests that there is indeed a strong community of students who are actively interested in learning more about healthcare innovation (e.g. the attendee ‘Demand’ for these events is substantial). These results combined with the neutral *Confidence* and *Intention* data suggest that there are actionable opportunities for policymakers and administrators to fulfill this demand and foster innovation. For example, the significantly positive correlation between *Confidence* and *Intent* ( **Figure 2**) suggests that funding for curricula designed to improve entrepreneurial confidence through skills workshops and seminars may, in-turn, increase student intent in pursuing innovative healthcare ventures. Furthermore, grant support for worthy educational experiences can encourage students to actively seek out additional experiences, courses, and self-directed learning in fields that drive their individual entrepreneurial curiosity and interest. Additionally, institutional support through scholarships and establishment of physical space for laboratories, incubators, and accelerators can aid in removal of financial barriers and promote a culture of innovation. Moreover, public and private academic institutional support for these Innovation Summit-type events provides a venue for participants to build their skills sets, test their innovative ideas, and network within a like-minded community.

A secondary goal of this work was to provide baseline metrics for future studies investigating trends of entrepreneurial characteristics in similar populations over time. As such, it is important to consider whether the metrics measured here sufficiently captured the entrepreneurial profile of the sampled population, or whether future studies should investigate additional metrics. The results of **Figure 3** show that combined, all the variables measured in this study result in linear models of *Intention* and *Confidence* with adjusted R squared values of 0.66 and 0.55, respectively. While these values are a marked improvement in captured variability compared to the simple pairwise linear regression models of **Figure 2**, they are still relatively low. One explanation is that this is because a simple linear model is not the best fit for the system at hand, which would warrant additional, more sophisticated analyses of larger data sets.

Alternatively, these results may suggest there are additional characteristics that were not accounted for in this study. As such, we would suggest that future studies include more detailed and segmented entrepreneurial metrics, perhaps by adding additional specific questions regarding each characteristic (for example: subcategories of confidence regarding different genres of entrepreneurial skills, such as securing funding, building a team, finding mentors, etc.).

The results of this work set a foundation for numerous additional studies. In particular, it would be worthwhile to capture direct measurements of the utility of Innovation Summit-type events by investigating how they affect entrepreneurial characteristics of participants. Such a study could be accomplished with a pre-and-post event survey using similar data collection methods to those in this study and would likely provide valuable information regarding how to best improve and tailor these events in the future. Additionally, continued demographic assessments at these events may help inform how well they are being marketed to-and connecting with-different student groups. Furthermore, studies investigating trends in entrepreneurial characteristics of a cohort of students who participate in multiple curricular events over time would likely produce useful and informative data about the role these events play in fostering entrepreneurial features.

### Limitations

This study is subject to potential limitations. Small sample size may have led to type II error, resulting in an inability to parse results by demographics or education level (e.g. graduate, medical, undergraduate) as well as an inability to look for significant difference in entrepreneurial characteristics between groups. Also, the largest subset of the sampled population was medical students. Despite the seemingly apparent increase in entrepreneurship amongst graduate students, medical students in particular are less likely to have a primary and immediate goal of starting a company given the prospect of residency training after graduation. This may have biased responses to questions such as “My professional goal is to become an entrepreneur,” and lowered the mean *Intention* category score ( **Appendix 2**).

In addition, results from this study may be difficult to generalize more broadly to students in different regions or in different, non-medical educational trajectories since respondents were exclusively registrants of a single Innovation Summit, which took place at a medical institution in New Jersey. Furthermore, sampling bias must be considered given that eager entrepreneurs who registered for the event may have been more likely to participate in the study due to their overall passion for the subject.

While the sample population in this study was relatively small, it is important to note that the reported demographics were considerably skewed towards early-to-mid twenties, Caucasian, males. Greater diversity of backgrounds and ideas is critical for maximal innovation in healthcare. Thus, observing similar trends in future studies would warrant additional research investigating the cause of these skewed demographics. Further demographic studies showing similar skews in age, race, and gender would warrant advocacy for additional funding and support of initiatives that encourage entrepreneurial interest across diverse student populations.

## Conclusion

In this study we sought to quantify, for the first time, the entrepreneurial intent and associated characteristics of undergraduate and graduate students from universities within a major corridor of healthcare innovation (e.g. the Northeast Corridor). Our findings constitute a lead-off point in further growing an existing culture of healthcare innovation and entrepreneurship in this region. These data also demonstrate the integrated nature of these characteristics, supporting the idea that investment that promotes one area, for example *Confidence,* would translate to increases in others such as *Intent.* Opportunities for expansion on the findings of this study are substantial and could detail trends within the larger entrepreneurial-medicine environment.

## Take Home Messages

• To make entrepreneurial-medicine instruction effective, medical educators must tailor these curricula to the entrepreneurial attitudes of students. As such, medical educators must be knowledgeable of the types of students that participate in these curricula.
• Healthcare students participating in a medical-entrepreneurship curriculum demonstrate significant *Interest* in an entrepreneurial career and perceive positive *Support* from their community and peers for entrepreneurial pursuits, yet are neutral in their *Confidence* and *Intent* to pursue an entrepreneurial venture.
• Entrepreneurial *Interest* was significantly, positively correlated with all three other characteristics ( *Intention*, *Confidence*, and *Support*). This suggests curricula-based efforts that foster *Interest* can bolster *Confidence* and *Intent.*
• *Confidence* and *Intent* are also positively correlated, suggesting that curricula designed to improve entrepreneurial *Confidence* through skills workshops and seminars may, in-turn, increase student *Intent* to pursue innovative healthcare ventures.

## Notes On Contributors

1. Alexander Watson: Alexander Watson is a medical student at Rutgers-Robert Wood Johnson Medical School (R-RWJMS) and a Rutgers Business School MBA graduate. He was president of the Biomedical Entrepreneurship Network (BEN) from 2016-2017 and contributor to the development of the Distinction in Medical Innovation and Entrepreneurship (DiMIE) at R-RWJMS.

2. Camden MacDowell: Camden MacDowell is a MD-PhD combined degree candidate at Rutgers-Robert Wood Johnson Medical School and Princeton University. He was on the executive board of the Biomedical Entrepreneurship Network (BEN) from 2016-2017 and also contributed to development of the Distinction in Medical Innovation and Entrepreneurship (DiMIE) at R-RWJMS.

3. Gregg Khodorov: Gregg Khodorov is a medical student at Rutgers-Robert Wood Johnson Medical School (R-RWJMS) and a Rutgers Business School MBA graduate. While Co-President of the Biomedical Entrepreneurship Network (BEN), Gregg coordinated and planned the first-ever BEN Health Innovation Summit, and served as a moderator for a panel on redesigning payer-provider models.

4. Julia Tartaglia: Julia Tartaglia is a medical student at Rutgers-Robert Wood Johnson Medical School. She has conducted research in sleep and cognition at the BIDMC Center for Sleep and Cognition. Julia served as co-President of the Biomedical Entrepreneurship Network and co-director of the BEN Health Innovation Summit.

5. Paul Weber, MD, RPh, MBA: Dr. Paul Weber is a licensed physician, pharmacist and MBA graduate with experience in academia, currently as an Associate Dean for CME at Rutgers-Robert Wood Johnson Medical School, Director for the Health Systems Science Curriculum Thread, and as an educator.

## Appendices

### Appendix 1: Survey Questions

#### Entrepreneurial Intention

Indicate your level of agreement with the following sentences from 1 (total disagreement) to 7 (total agreement).

a. Being an entrepreneur implies more advantages than disadvantages to me
b. A career as entrepreneur is attractive for me
c. If I had the opportunity and resources, I’d like to start a firm
d. Being an entrepreneur would entail great satisfactions for me
e. Among various options, I would rather be an entrepreneur

#### Entrepreneurial Support Network

If you decided to create a firm, would people in your close environment approve of that decision? Indicate from 1 (total disapproval) to 7 (total approval).

a. Your close family
b. Your friends
c. Your colleagues

#### Entrepreneurial Confidence

To what extent do you agree with the following statements regarding your entrepreneurial capacity? Value them from 1 (total disagreement) to 7 (total agreement).

a. To start a firm and keep it working would be easy for me
b. I am prepared to start a viable firm
c. I can control the creation process of a new firm
d. I know the necessary practical details to start a firm
e. I know how to develop an entrepreneurial project
f. If I tried to start a firm, I would have a high probability of succeeding

#### Entrepreneurial intention (EI)

Indicate your level of agreement with the following statements from 1 (total disagreement) to 7 (total agreement)

a. I am ready to do anything to be an entrepreneur
b. My professional goal is to become an entrepreneur
c. I will make every effort to start and run my own firm
d. I am determined to create a firm in the future
e. I have very seriously thought of starting a firm
f. I have the firm intention to start a firm some day

### Appendix 2: Individual Question Answers

**Table:**

Characteristic	Question	Mean	SD
Interest	Being an entrepreneur implies more advantages than disadvantages to me.	5.58	1.22
Interest	A career as an entrepreneur is attractive for me.	5.37	1.67
Interest	Being an entrepreneur would entail great satisfaction for me.	5.53	1.58
Interest	Among various options, I would rather be an entrepreneur.	4.42	1.61
Support	Your close family	5.79	0.92
Support	Your friends	5.89	1.05
Support	Your colleagues	5.74	1.24
Intention	I am ready to do anything to be an entrepreneur.	4.16	1.21
Intention	My professional goal is to become an entrepreneur.	3.37	1.38
Intention	I will make every effort to start and run my own firm.	4.16	1.54
Intention	I am determined to create a firm in the future.	3.26	1.73
Intention	I have very seriously thought of starting a firm.	4.00	1.97
Intention	I have the firm intention to start a firm some day.	4.11	1.29
Confidence	To start a firm and keep it working would be easy for me.	3.74	1.76
Confidence	I am prepared to start a viable firm.	3.58	1.98
Confidence	I can control the creation process of a new firm.	3.68	1.77
Confidence	I know the necessary practical details to start a firm.	4.16	1.98
Confidence	I know how to develop an entrepreneurial project.	4.47	2.14
Confidence	If I tried to start a firm, I would have a high probability of succeeding.	4.26	1.94
