# Supplementary material for: Assessing Entrepreneurial Characteristics of Healthcare Students Participating in an Entrepreneurial-Medicine Curriculum
Source: MedEdPublish (2016). 2018 Oct 16;7:101. Originally published 2018 May 15. [Version 2] doi: 10.15694/mep.2018.0000101.2 (PMC10699374; doi:10.15694/mep.2018.0000101.2)
Supplement: Peer review history

# Reviewer report: Reviewer response for version 2

**DOI:** 10.21956/mep.20221.r31317

**Published:** 2019-10-15

**Authors:** Trevor Gibbs

**Affiliations:** 1 AMEE

This review has been migrated. The reviewer awarded 4 stars out of 5 Not having reviewed the first paper, I did before I reviewed this second version. I enjoyed reading both and I think that the authors have addressed the concerns from the first review.Although I found the paper well written and important, I was at a slight loss to know if the authors confused somewhat the issues of innovation and entrepreneurialism, especially when applied to medical education and medical student development. I find students incredibly innovative in their thinking and doing and find that this is frequently knocked out of them during medical school, just as simple curiosity is. Not detracting from this issue however, I did feel that this was a paper that should be read by all curriculum developers.

Reviewer Expertise:

NA

No decision status is available

# Reviewer report: Reviewer response for version 2

**DOI:** 10.21956/mep.20221.r31316

**Published:** 2019-03-14

**Authors:** Richard Hays

**Affiliations:** 1 James Cook University

This review has been migrated. The reviewer awarded 4 stars out of 5 While I found this paper (and the original version) to be fascinating, two issues detract from its value. The first is that entrepreneurialism is a broad concept, yet this focus here is on a starting up a business. I am not sure that this captures adequately the scope for innovation and creative development within exisiting organisations (including the huge public health sector). The second concern is the low response rate, which limits severely any interpretation. However, medical educators need to think more about the concepts of innovation and development, as several registration bodies used similar terms to describe helping medical graduates achieve learning outcomes that shape the future and solve problems in the health care system.

Reviewer Expertise:

NA

No decision status is available

# Reviewer report: Reviewer response for version 2

**DOI:** 10.21956/mep.20221.r31315

**Published:** 2019-02-10

**Authors:** Ken Masters

**Affiliations:** 1 Sultan Qaboos University

This review has been migrated. The reviewer awarded 4 stars out of 5 This is a really useful article that addresses the business side of healthcare; an area often ignored in curricula, and - from personal experience with medical students – is often seen as less important than other components, especially clinical components.The authors have addressed the previous reviewer’s criticisms adequately. Pointedly, they have acknowledged the lost opportunity to develop a more direct tool aimed at medical students, but have also shown that, in balance, the tool was reasonably suitable to the purpose.It would be encouraging if the authors elected to take this further, develop a new tool, and then repeat the study with a new group of students. Their results, I am sure, would prove invaluable.

Reviewer Expertise:

NA

No decision status is available

# Reviewer report: Reviewer response for version 1

**DOI:** 10.21956/mep.19111.r27564

**Published:** 2018-08-20

**Authors:** Ariela Marshall

**Affiliations:** 1 Mayo Clinic

This review has been migrated. The reviewer awarded 3 stars out of 5 This is a fascinating article which is very timely in the current health care climate. I read it with interest and think that the authors have done a nice job surveying participants in the healthcare Innovation Summit about aspects of entrepreneurship. However, the major barrier I see is the use of a survey instrument which is validated only in the business world and meant for those who intend careers in business (not medicine). Further discussion below:Abstract: Some of the wording was awkward such as “significantly positively correlated” and could be rewritten as, “There were significant positive correlations between X and Y….”. Introduction: This was extremely well-worded and very helpful by way of background – both why the authors are interested in the topic as well as a good explanation of the Innovation Summit structure. May wish to include a brief discussion of the Millennial learner and how business/entrepreneurship are well-suited for the Millennial generation.Methodology: My major criticism is that the “validated” tool the authors use has been validated only in the business world and many of the questions do not seem applicable to those in medicine. The authors mention this as a limitation of the study, but my question is: why did the authors not rewrite/reframe/reword some of the questions to create their OWN survey tool which would be the first of its kind for assessment in medicine? I personally think that creation of a survey tool and piloting it with this group then provides the framework for validation in future studies and gives the authors real credibility as innovators in this area. I would much prefer to see modification of a validated tool – even if the study results presented here are not “validated” – than reliance on a tool that is not directly applicable to most medical practitioners.Discussion: This was nicely written and the point about institutional funding to allow promotion of a culture of innovation is well made. The standard limitations of small sample size, homogeneous population etc were appropriately addressed. However, the main point remains the lack of generalizability as I discussed above due to the business focused nature of the validated tool used for assessment. The authors should call for creation of a healthcare-entrepreneurial assessment tool and then create it themselves. This paves the way for future career development/opportunities in this area.

Reviewer Expertise:

NA

No decision status is available
